# Late Pregnancy Exposures to Disinfection By-products and Growth-Related Birth Outcomes

**DOI:** 10.1289/ehp.8282

**Published:** 2005-08-17

**Authors:** Alison F. Hinckley, Annette M. Bachand, John S. Reif

**Affiliations:** Department of Environmental and Radiological Health Sciences, Colorado State University, Fort Collins, Colorado, USA

**Keywords:** birth weight, disinfection by-products, epidemiology, haloacetic acids, pregnancy, preterm birth, trihalomethanes

## Abstract

Toxicologic studies have demonstrated associations between growth-related birth outcomes and exposure to high concentrations of disinfection by-products (DBPs), including specific tri-halomethane (THM) and haloacetic acid (HAA) chemical subspecies. Few prior investigations of DBPs have evaluated exposure during the third trimester of pregnancy, the time period of gestation when fetal growth may be most sensitive to environmental influences. We conducted a retrospective cohort study to examine the effects of exposure to THMs and HAAs during the third trimester and during individual weeks and months of late gestation on the risks for term low birth weight, intrauterine growth retardation, and very preterm and preterm births. The study population (*n* = 48,119) included all live births and fetal deaths occurring from January 1998 through March 2003 to women whose residence was served by one of three community water treatment facilities. We found evidence of associations between exposure to specific HAAs and term low birth weight as well as intrauterine growth retardation and for exposure to the five regulated HAAs (HAA5) and term low birth weight. Our findings suggest a critical window of exposure with respect to fetal development during weeks 33–40 for the effects of dibromoacetic acid and during weeks 37–40 for the effects of dichloroacetic acid. Adjustment for potential confounders did not affect the conclusions.

The chemical mixture of disinfection byproducts (DBPs) has not been fully characterized but is known to contain trihalomethanes (THMs), haloacetic acids (HAAs), haloacetonitriles, and other classes of chemicals, some of which are mutagenic or carcinogenic in laboratory animals (Nieuwenhuijsen et al. 2000). Total THMs (TTHMs) are the sum of the concentrations of the THM species chloroform, bromodichloromethane (BDCM), dibromochloromethane (DBCM), and bromoform. The five regulated HAAs (HAA5) include monochloroacetic acid (MCAA), dichloroacetic acid (DCAA), trichloroacetic acid (TCAA), monobromoacetic acid (MBAA), and dibromoacetic acid (DBAA). Concerns have been raised regarding the potential effects of by-products on reproductive outcomes, supported in part by the findings that some by-products cause reproductive and developmental toxicity in laboratory animals, albeit at doses much higher than those encountered by humans. In addition, exposure to DBPs has been associated with an increased risk of impaired fetal growth in several epidemiologic studies (Bove et al. 1995; Dodds et al. 1999; Gallagher et al. 1998; Kramer et al. 1992; Savitz et al. 1995; Wright et al. 2003).

The third trimester of pregnancy is considered the period of human fetal development during which fetal growth and birth weight are maximally sensitive to environmental influences (Kline et al. 1989). The third trimester lasts from approximately the 26th week of gestation to parturition, with the actual length of time dependent on the individual pregnancy. However, only a few prior investigations of DBPs have evaluated exposure during this period (Dodds et al. 1999; Gallagher et al. 1998; Savitz et al. 1995; Wright et al. 2003). Risks for adverse birth outcomes depend on the magnitude of exposure over critical time windows. Therefore, analyses over exposure windows that are too wide may bias risk estimates. Because the critical time period for the potential effects of DBP exposure on fetal growth is uncertain, the use of multiple, shorter exposure windows may provide less biased risk estimates (Hertz-Picciotto et al. 1996). The purpose of this study was to examine the effects of exposure to THMs and HAAs during the third trimester and during individual weeks and months of late gestation on the risks for term low birth weight, intrauterine growth retardation, and very preterm and preterm births.

## Materials and Methods

We conducted a retrospective cohort study in a large Arizona community served by three water treatment facilities. This community of more than half a million residents living in 24 ZIP codes is located adjacent to a major metropolitan area. Most water used by this community originates from surface water sources by means of the Salt River and Central Arizona projects.

The community was selected from the U.S. Environmental Protection Agency (EPA) Information Collection Rule database (U.S. EPA 1999) because the distribution systems displayed large temporal fluctuations (range, 7–81 μg/L) and low spatial variability in TTHM levels that permitted a natural experiment through intracommunity comparisons of exposures and outcomes. We determined spatial variability using the methods described by Hinckley et al. (2005). Briefly, we classified a facility as having low spatial variability if TTHM values measured at four points in the facility’s distribution system consistently fell, each season, within established boundaries for low, medium, and high exposure as based on concentration cut-points for TTHMs derived from prior epidemiologic studies of birth outcomes. The study population included all live births and fetal deaths for women whose residence was provided water by one of three facilities serving the community from January 1998 through December 2002. Table 1 summarizes the annual frequency of births in this population, as well as the distribution of TTHM and HAA5 concentrations by year over the period of the study. This study was approved by the human subjects institutional review boards of Colorado State University and the Arizona Department of Health Services.

Subjects were identified from Arizona birth records (*n* = 48,119) and were matched to a facility service area by residential ZIP code. In cases where two facilities shared the same distribution system, treatment facility employees identified service boundaries. Subjects who lived in ZIP codes that received water from more than one facility were excluded from the analysis. Maternal residence at birth was assumed to be the same as residence during the third trimester.

We estimated exposure from data obtained from each facility (facilities A–C) for the years 1998–2002. Total and individual THMs were measured quarterly during each of the 5 years, for each of the facilities. Facility A provided quarterly THM and HAA data for the entire study period, with monthly data available for 2001 and 2002. At facilities B and C, HAA5 data were available only for 2000 and 2002. Supplemental monthly and biweekly TTHM and HAA5 data were provided by facility B for 2000 and 2002, respectively. DBP concentrations were monitored at two to four locations within the distribution system of each facility. The quarterly and monthly data indicated the presence of very low levels of bromoform, MBAA, and MCAA; therefore, these chemicals were not included in the analyses. To estimate DBP values for specific study periods corresponding to months when no data were available, we performed a spline regression (Greenland 1998) for each water facility to impute the missing values using procedures similar to those used by investigators in Nova Scotia (Dodds et al. 1999; Dodds and King 2001; King et al. 2000). This nonlinear smoothing technique was applied to impute missing exposure data from existing data by generating a joined series of parabolic curve segments. HAA exposure data were not estimated before the year 2000 for facilities B and C.

Infant outcomes were identified through vital records. The date of last menstrual period was used to define the duration of gestation. We identified infants born at ≥37 completed weeks of gestation and weighing < 2,500 g as being term low birth weight. We evaluated this outcome only among term births to separate children with true growth retardation from babies that are small because of birth at a young gestational age. We identified case infants with intrauterine growth retardation as term or preterm babies that fell below the published value for the lowest 10th percentile of birth weights by race, ethnicity, and gestation age (Alexander et al. 1999). In this investigation, term low birth weight and intrauterine growth retardation were not mutually exclusive, and cases born at term may have been included in both outcome groups. Because published values for the lowest 10th percentile of birth weights were not available for extreme gestational ages, births before 23 weeks’ gestation were excluded from intrauterine growth retardation analyses for Caucasians, African Americans, and Hispanics, and births before 29 weeks’ gestation were excluded for Native Americans (Alexander et al. 1999). For intra-uterine growth retardation and term low birth weight, estimated monthly DBP exposures were averaged over the third trimester. Additionally, for DBPs associated with intrauterine growth retardation or term low birth weight, we averaged and evaluated exposure during specific time windows, corresponding to gestation weeks 25–28, 29–32, 33–36, 37–40, and 41–44, using monthly DBP concentrations.

Preterm births were defined as infants born at < 37 completed weeks of gestation. Very preterm births were defined as a birth occurring before 32 completed weeks of gestation (Martin et al. 2002). Because preterm birth outcomes are defined by time length of gestation, it was inappropriate to evaluate exposure averaged over the third trimester. Preterm births have shorter gestation lengths than the typical comparison group (term births), increasing the potential for bias (Hertz-Picciotto et al. 1996; Hinckley 2003; Hinckley et al. 2002). Therefore, for preterm and very preterm births, we evaluated exposure to DBPs only for the specific gestation week intervals mentioned above.

We abstracted information on potential confounders from birth records. These variables included maternal age, race, ethnicity, education, parity, smoking, and the Kessner index (a measure of prenatal care adequacy) (Kotelchuck 1994). By comparing the outcomes over different exposure time windows within a single community, we attempted to control for potential residual confounders that could not be evaluated individually.

We calculated tertiles of DBP concentrations using the species-specific data from all three water treatment regions in analyses for potential associations with each birth outcome. We used stratified chi-square and logistic regression analyses to evaluate the associations among demographic variables, exposure variables, and adverse birth outcomes. In addition, all covariates significantly associated with growth outcomes at the < 0.20 level in univariate analyses were retained for inclusion in multivariable analyses. After adjustment for potential confounders, we calculated odds ratios (ORs) and 95% confidence intervals (CIs) for the relationships between all individual THM species and growth and preterm birth outcomes. For gestation week and third-trimester analyses, a multivariate logistic regression model containing all individual HAAs as continuous variables was used to evaluate the possible relationship between individual HAAs in increasing the risk of growth-related outcomes. A similar model was not created for individual THMs because there was no evidence of any associations with growth-related outcomes.

## Results

Table 2 summarizes characteristics of subjects and frequency of intrauterine growth retardation, term low birth weight, and preterm and very preterm births. Most mothers were white, non-Hispanic, nulliparous women with some college education. Most mothers received adequate prenatal care, and < 10% smoked during pregnancy. Subjects were excluded if there was no date for last menstrual period or no estimated date of conception. The estimated date of conception was used to estimate the last menstrual period when data on last menstrual period were missing or considered extreme (> 44 weeks before the birth date). The results were not different when using last menstrual period, estimated date of conception, or a combination of both methods; therefore, we used the combined method to minimize the number of subjects lost for this reason (*n* = 42).

The ORs and 95% CIs for intrauterine growth retardation and term low birth weight and exposure to DBPs during the third trimester are shown in Table 3. We found no evidence of an association with either outcome for exposure to TTHMs or specific brominated and chlorinated THMs. We also found no association between exposure to HAA5 and intrauterine growth retardation. The second and third tertiles of exposure to HAA5 showed evidence of a weak association with term low birth weight [OR = 1.26 (95% CI, 0.96–1.65), and OR = 1.25 (95% CI, 0.96–1.64), respectively] compared with referent exposure levels.

Exposures to the highest tertiles of DCAA and TCAA were associated with an increased risk of intrauterine growth retardation [OR = 1.28 (95% CI, 1.08–1.51), and OR = 1.19 (95% CI, 1.01–1.41), respectively]. DCAA and TCAA were also associated with intrauterine growth retardation when analyzed as continuous variables. Weak associations were found for exposure to the highest tertile of DBAA and DBAA analyzed as a continuous variable, although the 95% CIs for those results all included 1.0. Analyses of intrauterine growth retardation were adjusted for parity, smoking, maternal education, and Kessner index.

The risk of term low birth weight was increased (OR = 1.49; 95% CI, 1.09–2.04) among women exposed to average DBAA concentrations of ≥5 μg/L during the third trimester compared with those who were exposed to the referent category of < 4 μg/L. Continuous (unit) increases in average exposure to DBAA also indicated a weak association with term low birth weight (OR = 1.17; 95% CI 1.03–1.32). Analyses of term low birth weight were adjusted for maternal age, parity, education, race, ethnicity, smoking, and Kessner index.

Table 4 presents ORs and 95% CIs for exposure to HAA5 and individual HAAs over specific gestation time windows for intrauterine growth retardation and term low birth weight. Because the potential for bias due to averaging was reduced when examining shorter time intervals, exposure values were generally slightly higher or slightly lower over the specific gestation week intervals than over the third trimester. In analyses for intrauterine growth retardation, small increases in risk were observed for DBAA concentrations ≥5 μg/L (OR = 1.15; 95% CI, 0.98–1.35) and for DBAA analyzed as a continuous variable (OR = 1.06; 95% CI, 1.01–1.12) over gestation weeks 25–28. The largest risk was observed with exposure to DCAA ≥8 μg/L (OR = 1.27; 95% CI, 1.02–1.59) during gestation weeks 37–40. In addition, an increased risk was observed for exposure to moderate concentrations of TCAA (OR = 1.58; 95% CI, 1.02–2.46) and DCAA (OR = 1.51; 95% CI, 0.98–2.32) during gestation weeks 41–44, but the risk estimates were lower at higher levels of estimated exposure.

Exposure to DBAA was associated with an increase in risk for term low birth weight in analyses by gestation week. Between gestation weeks 33 and 36, the second and third tertiles of exposure to DBAA showed evidence of a dose-dependent trend [OR = 1.29 (95% CI, 0.94–1.79), and OR = 1.49 (95% CI, 1.10–2.02), respectively] compared with referent exposure levels. Similarly, moderate exposure to DBAA between gestation weeks 37 and 40 was associated with an increased risk for term low birth weight (OR = 1.38; 95% CI, 1.02–1.86).

No associations were observed between preterm or very preterm birth and exposure to TTHMs, HAA5, or specific DBPs during any gestation week interval. ORs for the associations between individual HAAs and term low birth weight and intrauterine growth retardation were not affected by inclusion of other HAAs in the logistic regression model.

## Discussion

Reduced fetal weight is one of the most consistent developmental effects observed with exposure to high concentrations of DBPs in laboratory animals (Nieuwenhuijsen et al. 2000). The biologic mechanisms for DBP-induced growth retardation are not well understood. In animal studies, reductions in birth weight have been commonly described after exposure to THMs, especially chloroform (Murray et al. 1979; Ruddick et al. 1983; Schwetz et al. 1974; Thompson et al. 1974). Two studies by Smith et al. (1989, 1992) found reductions in rat pup body weight after exposure to DCAA and TCAA. Recently, Christian et al. (2001) found that DBAA administration (of 250, 500, and 1,000 mg/L) was associated with exposure-related decreases in rat pup body weight. This effect, however, was thought to be due to reduced parental water consumption.

The epidemiologic evidence for an association between exposure to THMs and indicators of fetal growth is relatively sparse and inconsistent, and few studies have investigated this relationship with respect to HAAs. Four prior epidemiologic studies have evaluated exposure to total and individual THMs in relation to intrauterine growth retardation. In a study by Kramer et al. (1992), a dose-related trend was observed for intrauterine growth retardation at the 5th percentile for exposure to chloroform ≥10 μg/L and BDCM ≥4 μg/L, with ORs of 1.8 (95% CI, 1.1–2.9) and 1.7 (95% CI, 0.9–2.9), respectively. Bove et al. (1995) also found an increased risk of intrauterine growth retardation (adjusted OR = 1.50; 90% CI, 1.19–1.86) with exposure to TTHMs > 100 μg/L during pregnancy. In a Massachusetts cohort, Wright et al. (2003) found increased risk of intra-uterine growth retardation (10th percentile) for mean exposures to TTHMs > 80 μg/L throughout pregnancy (adjusted OR = 1.14; 95% CI, 1.02–1.26) and during the second trimester (adjusted OR = 1.13; 95% CI, 1.03–1.24). However, Dodds et al. (1999) found no association between intrauterine growth retardation (10th percentile) and TTHM exposure ≥100 μg/L in a large cohort of Nova Scotia women.

Three studies have evaluated term low birth weight and exposure to TTHMs. Gallagher et al. (1998) found an adjusted OR of 5.9 (95% CI, 2.0–17.0) for term births, although only six cases were analyzed. Bove et al. (1995) also observed a positive, but smaller, association between TTHM exposures averaged over the entire pregnancy and term low birth weight with an OR of 1.42 (50% CI, 1.22–1.65). In a study by Wright et al. (2003), no associations were reported between term low birth weight and trimester-specific exposures or entire pregnancy exposures to TTHMs. Six studies have evaluated preterm birth or very preterm birth; none found a significant relationship with DBPs (Bove et al. 1995; Dodds et al. 1999; Gallagher et al. 1998; Kramer et al. 1992; Savitz et al. 1995; Wright et al. 2003).

As a group, these studies differed in their selection of a referent group for exposure, in their ability to control for potential confounding, and in their assessment of exposure during the third trimester or late stages of pregnancy. To evaluate the relationship between TTHMs and growth-related birth outcomes, Bove et al. (1995) averaged quarterly TTHM concentrations over each subject’s entire pregnancy. In a study of miscarriage, low birth weight and preterm delivery in North Carolina, Savitz et al. (1995) assigned exposure by using the quarterly value nearest the 28th week of pregnancy. Gallagher et al. (1998) used the median of all quarterly measurements taken during the third trimester. For children born in the second or third month of the quarter, Wright et al. (2003) used the average quarterly values for the third trimester; children born in the first month of the quarter were assigned the preceding quarterly averages. In Nova Scotia, Dodds et al. (1999) used linear regression of quarterly data to estimate average exposures during the last 3 months of pregnancy. Our method of assigning exposure included estimating some periodic study time exposures using a spline regression based on quarterly sampling values. Further, all data were interpolated from month midpoint and converted to ordinal study time, to better align with gestation time (Yang et al. 2005). This regression method, which is similar to that used in the Nova Scotia studies, permitted estimation of exposure for time periods when data were missing or when sampling was not performed. We performed a sensitivity analysis by systematically repeating the spline regression with varying subsets of exposure data. By this method, we found that the model consistently predicted existing data points to within ± 5%. However, the spline regression technique requires additional validation in other distribution systems.

Our study is the first to examine associations between exposures to specific HAAs and impaired fetal growth. We found evidence of associations between exposure to specific HAAs and term low birth weight and intra-uterine growth retardation. The second and third tertiles of exposure to HAA5 were also associated with a small increase in risk for term low birth weight when evaluated over the third trimester (Table 3). The increased risk in the second tertile did not seem to be due to a higher risk from DBAA, DCAA, or TCAA. HAA5 concentration is currently regulated in the United States, but concentrations of specific HAAs are not. Our findings suggest a critical window of exposure during weeks 33–40 for the effects of DBAA on fetal development. To our knowledge, this is the first time that DBAA has been investigated in an epidemiologic study of developmental outcomes. Studies of exposure to HAAs are relatively new, and none have been performed in communities where DBAA concentrations in drinking water were above detection (King et al. 2005; Wright et al. 2004). In this investigation, the levels of DBAA were well above the 90th percentile concentrations reported by the U.S. EPA (1998).

We also observed evidence of an association between intrauterine growth retardation and exposure to chlorinated HAAs during specific critical time windows of gestation, with modest increases in risk for third-trimester exposure to DCAA and slightly lower estimates for TCAA. When analyzed as continuous variables, exposure to DCAA and TCAA also showed slight increases in risk of intrauterine growth retardation between weeks 29 and 40 of gestation. The risk estimates remained consistent during the gestation week windows comprising this time period.

Our study is the first to examine exposure to DBPs during specific gestation week intervals of exposure. In previous studies, exposure for fetal development was usually averaged over the longer third-trimester window. Averaging a variable exposure over longer time periods such as the third trimester is likely to introduce misclassification over the critical time periods and lead to biased risk estimates (Hertz-Picciotto et al. 1996). However, for the highest level of exposure to DBAA, we observed the same OR for exposure averaged over the third trimester as for exposure averaged over gestation weeks 33–36. The CIs were narrower for DBAA exposure during weeks 33–36 than for the entire third trimester, reflecting increased precision due to the slightly larger sample population retained for analysis of gestation week intervals. Because the third trimester is a longer time period, it is more likely to fall outside of the study initiation and termination (or beginning and end) date than are single week-long periods (Hinckley 2003; Hinckley et al. 2002).

Windows of exposure have been historically important in epidemiologic investigations of thalidomide, retinoic acid (vitamin A), maternal rubella, and radiation (O’Rahilly and Muller 2001). For exposures during the first 2 weeks of gestation, few congenital abnormalities are observed because the teratogen either damages most cells, resulting in cell and embryonic death, or affects only a few cells that can be repaired without resultant birth defects (Moore and Persaud 1998). After the first 2 weeks, the tissue or organ that is most susceptible to malformation is the part undergoing critical development when the teratogen is active. Exposures that occur later in gestation have a less drastic effect and are thought to primarily affect fetal growth.

The strengths of this study include the large number of birth records, high quantity of exposure data (including some biweekly data), and the ability to evaluate multiple time periods of exposure to specific THMs and HAAs. By comparing subjects within the same community with respect to exposure levels, we may have reduced potential residual confounding. We also selected this community to minimize misclassification due to spatial variability within the distribution systems (Hinckley et al. 2005).

Our study was limited by the use of birth records to ascertain individual exposure information. Maternal residence was identified from birth records to assign the appropriate water service, but residential mobility during pregnancy may have introduced exposure misclassification. Potential exposure misclassification could also have resulted from lack of information regarding exposures from inhalation or dermal exposure from showering, bathing, and washing. Exposure estimates were based on distribution system DBP concentrations and did not account for variability in personal habits affecting ingestion, such as the use of bottled water (Zender et al. 2001). Finally, exposure misclassification could have resulted from exposures outside the service area (e.g., at work) of the designated water treatment system.

In summary, despite toxicologic evidence of growth retardation after exposure to DBPs, few human studies have been conducted on this relationship. The pervasive nature of the exposure suggests that even small effects may be important. This work explored this relationship using seasonal variability and intracommunity comparisons to define a natural experiment. We improved on previous exposure assessments by considering total and individual THMs and HAAs, and multiple time periods of exposure in late gestation. Further studies are needed to confirm our observations for DBAA, TCAA, and DCAA as well as other relationships between DBPs and growth outcomes.

## Figures and Tables

**Table 1:** Distribution of births/fetal deaths and TTHM and HAA5 concentrations [mean (range)] by year for the Arizona study community.

Year	No. of births/fetal deaths	TTHM concentrations (μg/L)	HAA5 concentrations (μg/L)
1998	9,117	56.9 (30.1–80.8)	31.8 (9.8–48.9)^a^
1999	9,571	45.8 (15.7–71.3)	20.6 (13.9–25.2)^a^
2000	9,976	48.9 (27.8–65.9)	17.0 (7.5–25.7)
2001	10,149	46.1 (16.6–68.0)	14.4 (4.2–22.3)^a^
2002	9,306	43.4 (7.8–70.3)	13.7 (3.1–23.0)

a Based on data from one facility.

**Table 2:** Characteristics of subjects and frequency of intrauterine growth retardation, term low birth weight, and preterm births [*n* (%)].

Characteristic	Study population (% of total)	Intrauterine growth retardation^a^	Term low birth weight	Very preterm birth	Preterm birth
Total births (% of total)	48,119 (100)	4,346 (9.5)	1,010 (2.1)	564 (1.2)	4,008 (8.3)
Maternal age (years)					
< 20	5,609 (11.7)	671 (15.4)	150 (14.9)	100 (17.7)	527 (13.1)
20–29	28,366 (59.0)	2,536 (58.4)	573 (56.7)	299 (53.0)	2,190 (54.6)
≥30	14,076 (29.3)	1,135 (26.1)	285 (28.2)	164 (29.1)	1,281 (32.0)
Unknown	68 (0.1)	4 (0.1)	2 (0.2)	1 (0.2)	10 (0.3)
Maternal race					
Caucasian	43,026 (89.4)	4,085 (94.0)	844 (83.6)	493 (87.4)	3,490 (87.1)
African American	1,347 (2.8)	114 (2.6)	52 (5.1)	41 (7.3)	173 (4.3)
Native American	1,715 (3.6)	147 (3.4)	33 (3.3)	7 (1.2)	146 (3.6)
Other	1,701 (3.5)	—	64 (6.3)	14 (2.5)	151 (3.8)
Unknown	330 (0.7)	—	17 (1.7)	9 (1.6)	48 (1.2)
Maternal ethnicity					
Non-Hispanic	30,803 (64.0)	2,741 (63.1)	623 (61.7)	349 (61.9)	2,575 (64.2)
Hispanic	15,140 (31.5)	1,453 (33.4)	340 (33.7)	191 (33.9)	1,228 (30.6)
Unknown	2,176 (4.5)	152 (3.5)	47 (4.7)	24 (4.2)	205 (5.1)
Maternal education					
≥1 year of college	22,435 (46.6)	1,769 (40.7)	391 (38.7)	217 (38.5)	1,779 (44.4)
High school graduate	13,427 (27.9)	1,266 (29.1)	281 (27.8)	171 (30.3)	1,139 (28.4)
< 12th grade	11,172 (23.2)	1,220 (28.1)	293 (29.0)	153 (27.1)	970 (24.2)
Unknown	1,085 (2.3)	91 (2.1)	45 (4.5)	23 (4.1)	120 (2.99)
Parity					
0	18,886 (39.3)	2,023 (46.5)	440 (43.6)	249 (44.1)	1,560 (39.9)
1	14,341 (29.8)	1,180 (27.2)	291 (28.8)	165 (29.3)	1,121 (28.0)
2	8,348 (17.4)	653 (15.0)	158 (15.6)	70 (12.4)	682 (17.0)
3	3,728 (7.8)	282 (6.5)	67 (6.6)	40 (7.1)	342 (8.5)
≥4	2,724 (5.7)	198 (4.6)	53 (5.2)	37 (6.5)	285 (7.1)
Unknown	92 (0.2)	10 (0.2)	1 (0.0)	3 (0.5)	18 (0.5)
Prenatal care (Kessner index)					
Adequate	36,271 (75.4)	3,059 (70.4)	663 (65.6)	350 (62.1)	2,604 (65.0)
Intermediate	9,117 (19.0)	941 (21.7)	243 (24.1)	124 (22.0)	948 (23.7)
Inadequate	2,731 (5.7)	346 (8.0)	104 (10.3)	90 (16.0)	456 (11.4)
Maternal smoking					
No	44,139 (91.7)	3,741 (86.1)	856 (84.7)	484 (85.8)	3,527 (88.0)
Yes	3,409 (7.1)	540 (12.4)	136 (13.4)	71 (12.6)	417 (10.4)
Unknown	571 (1.2)	65 (1.5)	18 (1.8)	9 (1.6)	64 (1.6)
Maternal alcohol					
No	47,176 (98.0)	4,233 (97.4)	976 (96.6)	547 (97.0)	3,906 (97.5)
Yes	291 (0.6)	39 (0.9)	11 (1.1)	5 (0.9)	29 (0.7)
Unknown	652 (1.4)	74 (1.7)	23 (2.3)	12 (2.1)	73 (1.8)

a Does not include births at < 23 weeks’ gestation for Caucasians, African Americans, and Hispanics or births at less than 29 weeks’ gestation for Native Americans. Does not include Asian or “other” births. For intrauterine growth retardation analyses, total number of births in data set = 41,682.

**Table 3:** ORs and 95% CIs for the association between exposure to DBPs averaged over the entire third trimester and intrauterine growth retardation and term low birth weight.

	Intrauterine growth retardation^a^		Term low birth weight^b^	
DBP (μg/L)	Cases (*n*)	OR (95% CI)	Cases (*n*)	OR (95% CI)
TTHMs (*n*)^c^	39,954		38,096	
< 40	1,208	—	269	—
40–53	1,198	0.98 (0.90–1.07)	284	1.06 (0.89–1.25)
≥53	1,354	1.09 (1.00–1.18)	306	1.11 (0.94–1.31)
Continuous	3,760	1.00 (1.00–1.01)	859	1.00 (1.00–1.01)
Chloroform				
< 10	1,216	—	265	—
10–16	1,258	1.02 (0.94–1.11)	312	1.18 (1.00–1.39)
≥16	1,286	1.01 (0.93–1.10)	282	1.04 (0.88–1.23)
Continuous	3,760	1.00 (1.00–1.01)	859	1.00 (1.00–1.01)
BDCM				
< 13	1,251	—	274	—
13–18	1,173	0.93 (0.85–1.01)	290	1.05 (0.89–1.24)
≥18	1,336	1.03 (0.95–1.12)	295	1.04 (0.88–1.23)
Continuous	3,760	1.00 (1.00–1.01)	859	1.00 (0.99–1.02)
DBCM				
< 12	1,288	—	286	—
12–16	1,164	0.96 (0.89–1.05)	269	1.00 (0.84–1.18)
≥16	1,308	1.01 (0.94–1.10)	304	1.05 (0.89–1.24)
Continuous	3,760	1.01 (1.00–1.01)	859	1.01 (0.99–1.02)
HAA5 (*n*)	14,350		13,981	
< 15	462	—	97	—
15–19	466	1.00 (0.87–1.15)	124	1.26 (0.96–1.65)
≥19	513	1.08 (0.94–1.23)	126	1.25 (0.96–1.64)
Continuous	1,441	1.0 (1.00–1.01)	347	1.01 (1.00–1.02)
DBAA (*n*)^d^	9,576		9,312	
< 4	322	—	70	—
4–5	301	1.04 (0.88–1.23)	71	1.01 (0.72–1.41)
≥5	347	1.12 (0.95–1.32)	103	1.49 (1.09–2.04)
Continuous	970	1.05 (0.98–1.12)	244	1.17 (1.03–1.32)
DCAA				
< 6	268	—	76	—
6–8	332	1.15 (0.97–1.36)	81	1.04 (0.75–1.43)
≥8	370	1.28 (1.08–1.51)	87	1.10 (0.80–1.50)
Continuous	970	1.05 (1.02–1.09)	244	1.02 (0.96–1.08)
TCAA				
< 4	277	—	73	—
4–6	309	1.00 (0.84–1.18)	81	0.94 (0.68–1.30)
≥6	384	1.19 (1.01–1.41)	90	1.00 (0.73–1.37)
Continuous	970	1.04 (1.02–1.07)	244	1.01 (0.96–1.05)

a Adjusted for parity, education, smoking, and Kessner index.

b Adjusted for maternal age, parity, education, race, ethnicity, smoking, and Kessner index.

c Sample sizes used in analysis of chloroform, BDCM, and DBCM were equal for third-trimester analyses.

d Sample sizes used in analysis of DBAA, DCAA, and TCAA were equal for third-trimester analyses.

**Table 4:** ORs (95% CIs) for term low birth weight and intrauterine growth retardation by gestation week (GW) according to level of DBP exposure.

DBP (μg/L)	GW 25–28	GW 29–32	GW 33–36	GW 37–40	GW 41–44
Intrauterine growth retardation^a^					
HAA5 (*n*)	14,350	14,953	15,414	14,929	2,634
< 14	—				
14–19	1.02 (0.89–1.17)	1.00 (0.86–1.13)	0.93 (0.81–1.06)	0.99 (0.86–1.13)	1.22 (0.86–1.72)
≥19	1.12 (0.98–1.29)	1.11 (0.98–1.27)	1.00 (0.88–1.14)	0.98 (0.85–1.13)	0.91 (0.63–1.33)
Continuous	1.01 (1.00–1.01)	1.01 (1.00–1.01)	1.00 (0.99–1.01)	1.00 (0.99–1.01)	1.00 (0.98–1.01)
DBAA (*n*)^b^	9,576	10,302	10,945	10,875	1,913
< 3	—				
3.5–5	1.00 (0.84–1.18)	0.97 (0.82–1.14)	1.14 (0.97–1.34)	0.95 (0.81–1.12)	1.23 (0.82–1.85)
≥5	1.15 (0.98–1.35)	1.06 (0.91–1.24)	1.08 (0.93–1.27)	0.90 (0.77–1.06)	0.91 (0.60–1.40)
Continuous	1.06 (1.01–1.12)	1.03 (0.99–1.08)	1.01 (0.96–1.06)	0.99 (0.94–1.04)	0.97 (0.85–1.10)
DCAA					
< 6	—				
6–8	1.00 (0.85–1.18)	1.06 (0.90–1.25)	0.92 (0.78–1.08)	1.00 (0.85–1.18)	1.51 (0.98–2.32)
≥8	1.04 (0.85–1.29)	1.11 (0.90–1.35)	1.03 (0.84–1.26)	1.27 (1.02–1.59)	1.41 (0.85–2.33)
Continuous	1.02 (0.99–1.04)	1.03 (1.01–1.05)	1.03 (1.01–1.05)	1.03 (1.01–1.05)	1.03 (0.98–1.08)
TCAA					
< 4	—				
4–6	0.96 (0.81–1.14)	1.05 (0.89–1.24)	0.91 (0.78–1.07)	1.12 (0.95–1.32)	1.58 (1.02–2.46)
≥6	1.01 (0.86–1.19)	1.15 (0.98–1.34)	1.07 (0.92–1.24)	1.15 (0.98–1.35)	1.48 (0.96–2.31)
Continuous	1.01 (1.00–1.03)	1.02 (1.01–1.04)	1.03 (1.01–1.05)	1.02 (1.01–1.04)	1.02 (0.98–1.06)
Term low birth weight^c^					
HAA5 (*n*)	13,981	14,593	15,195	15,780	2,755
< 14	—				
14–19	0.91 (0.69–1.18)	1.04 (0.79–1.36)	1.04 (0.80–1.36)	1.12 (0.87–1.44)	0.74 (0.28–1.98)
≥19	1.05 (0.81–1.36)	1.30 (1.00–1.69)	1.20 (0.93–1.55)	1.08 (0.83–1.40)	1.03 (0.39–2.72)
Continuous	1.01 (0.99–1.02)	1.01 (1.00–1.02)	1.00 (1.01–1.02)	1.00 (0.99–1.02)	0.99 (0.95–1.04)
DBAA (*n*)^b^	9,312	10,043	10,778	11,484	2,003
< 3	—				
3.5–5	0.97 (0.69–1.35)	1.05 (0.77–1.45)	1.29 (0.94–1.79)	1.38 (1.02–1.86)	0.99 (0.34–2.85)
≥5	1.16 (0.86–1.58)	1.18 (0.87–1.61)	1.49 (1.10–2.02)	1.22 (0.90–1.66)	0.38 (0.10–1.43)
Continuous	1.03 (0.94–1.12)	1.07 (0.98–1.17)	1.11 (1.01–1.21)	1.10 (1.01–1.20)	0.88 (0.62–1.23)
DCAA					
< 6	—			—	
6–8	0.80 (0.58–1.09)	0.84 (0.61–1.15)	0.94 (0.69–1.27)	0.98 (0.74–1.32)	0.45 (0.13–1.60)
≥8	0.87 (0.64–1.18)	0.99 (0.74–1.32)	0.98 (0.73–1.30)	0.91 (0.68–1.21)	0.93 (0.33–2.57)
Continuous	1.01 (0.97–1.05)	1.02 (0.98–1.06)	1.00 (0.96–1.04)	1.01 (0.97–1.05)	0.94 (0.81–1.09)
TCAA					
< 4	—				
4–6	0.79 (0.57–1.09)	0.91 (0.67–1.24)	1.05 (0.77–1.42)	1.15 (0.86–1.53)	0.40 (0.11–1.30)
≥6	0.89 (0.66–1.20)	0.98 (0.73–1.33)	1.05 (0.78–1.41)	0.93 (0.69–1.25)	0.64 (0.23–1.79)
Continuous	1.01 (0.98–1.05)	1.01 (0.98–1.04)	1.00 (0.96–1.03)	1.00 (0.96–1.03)	0.93 (0.80–1.09)

a Adjusted for parity, education, smoking, and Kessner index.

b Sample sizes used in analysis of DBAA, DCAA, and TCAA were equal for each gestation age interval.

c Adjusted for maternal age, parity, education, race, ethnicity, smoking, and Kessner index.
